# Correction: Generation and Characterization of a Novel Mouse Embryonic Stem Cell Line with a Dynamic Reporter of Nanog Expression

**DOI:** 10.1371/journal.pone.0101028

**Published:** 2014-06-20

**Authors:** The PLOS ONE Staff

## Notice of Republication

This article was republished on May 23, 2014, due to the exclusion of Figure 6 from the PDF. The publisher apologizes for the error. The originally published, uncorrected article and the republished, corrected article are provided here for reference.

## Supporting Information


**File S1.** Originally published, uncorrected article.
(PDF)

**File S2.** Republished, corrected article.
(PDF)
