# Supplementary figures and images for: Generation and Characterization of a Novel Mouse Embryonic Stem Cell Line with a Dynamic Reporter of Nanog Expression
Source: PLoS One. 2013 Mar 19;8(3):e59928. doi: 10.1371/journal.pone.0059928 (PMC3602340; doi:10.1371/journal.pone.0059928)

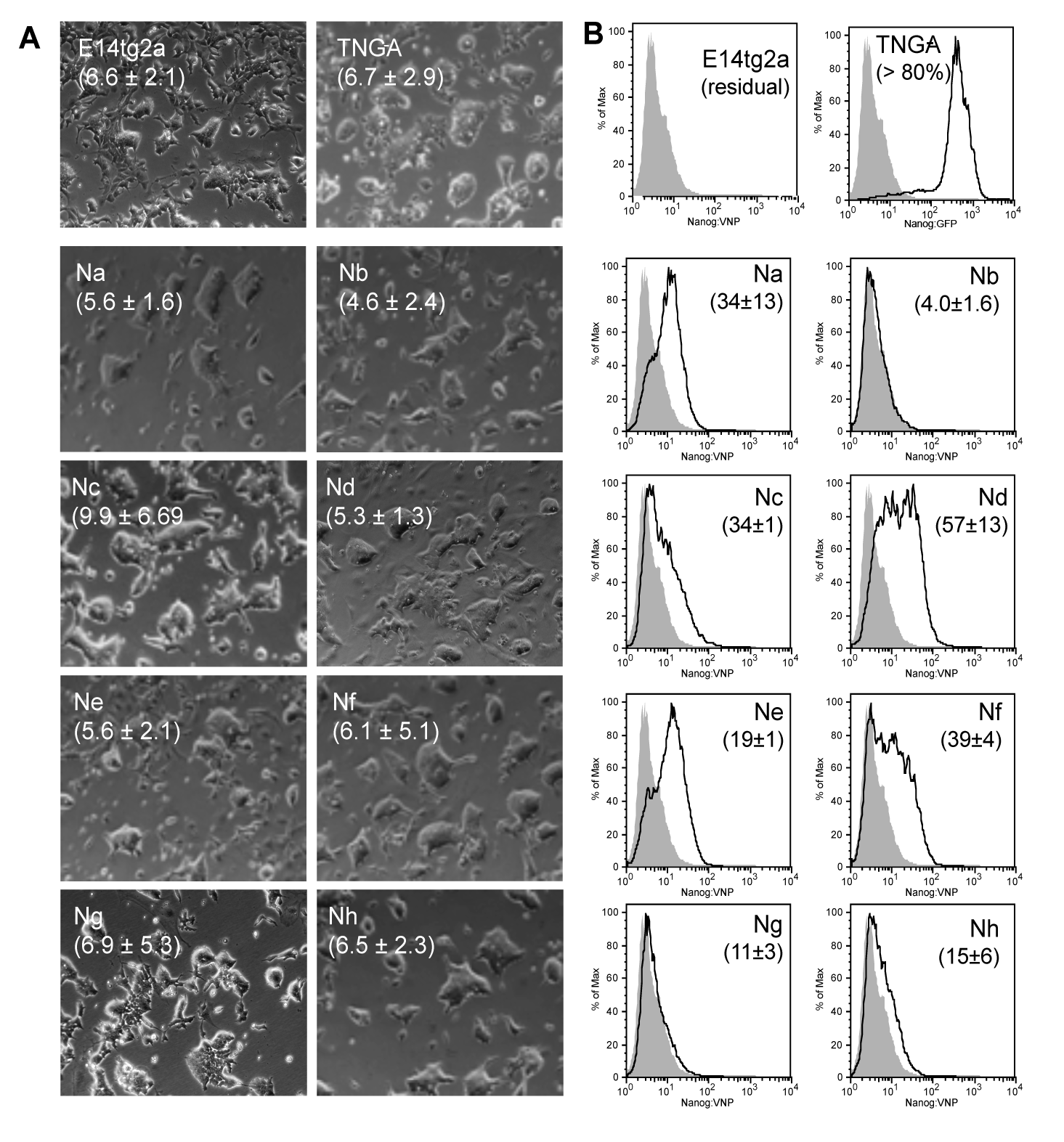

Supplement: Figure S1 — ES cell lines characterization: E14tg2a, TNG-A and transgenic clones. (A) Bright field images of E14tg2a, TNG-A and BAC-transgenic clones grown in serum/LIF conditions. Cell growth, measured as fold increase, is shown in brackets. (B) Representative FC histograms of VNP expression for E14tg2a (negative control, grey peak), TNG-A and transgenic clones (black lines) grown in serum/LIF. Reporters expression averages and standard deviations of at least 2 independent experiments are depicted on each graph for the corresponding cell line. (TIF) [file pone.0059928.s001.tif]

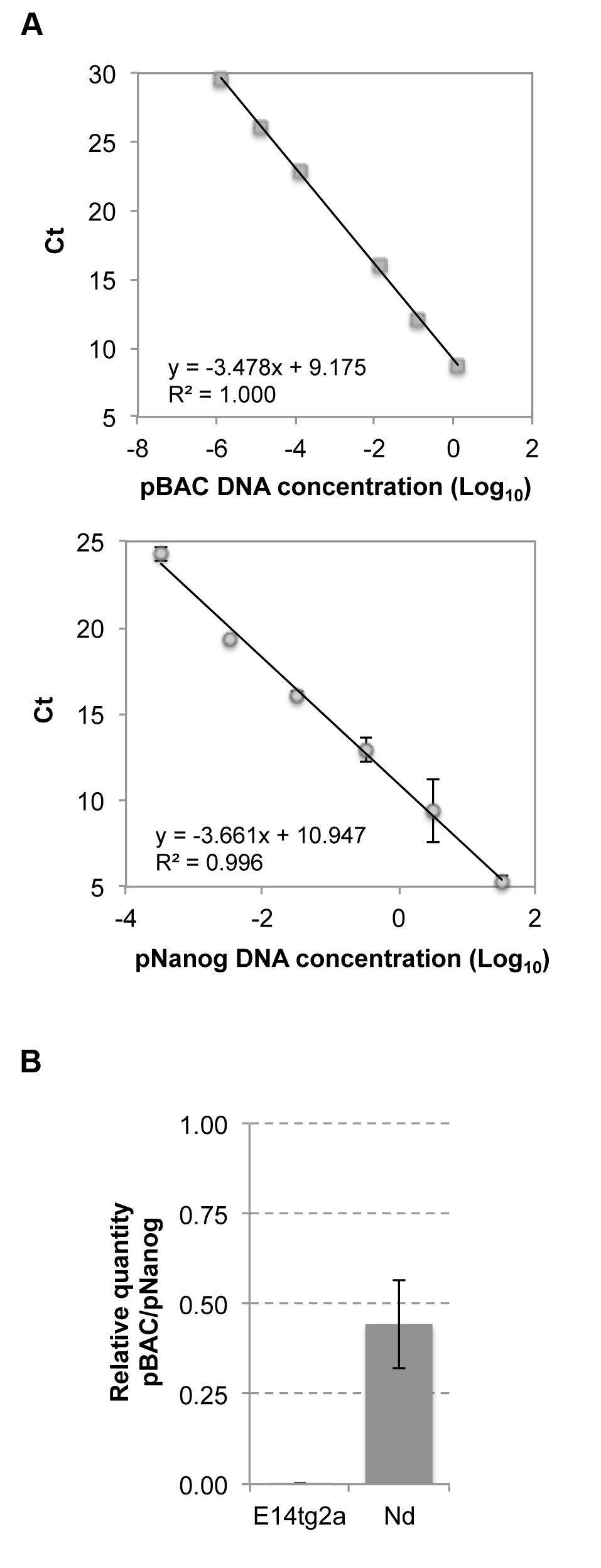

Supplement: Figure S2 — Copy number of integrated BAC in Nd ES cells. (A) Relative quantities of BAC (pBAC) and of Nanog (pNanog) were measured on a dilution series of DNA plasmids containing each sequence. Threshold cycle (Ct) values were plotted against log-transformed concentrations of plasmid DNA (n = 2). Trend lines were inserted and used to obtain values for slope (y = slope.x+y-intercept) and correlation coefficients (R2). (B) Quantities of pBAC relative to pNanog were determined for the wild-type E14tg2a ES cell line and for the Nd ES cell clone (n = 2). Plot shows that, as expected, no pBAC integration exists in wild-type ES cells, while the Nd clone has one copy of pBAC per two of pNanog. (TIF) [file pone.0059928.s002.tif]

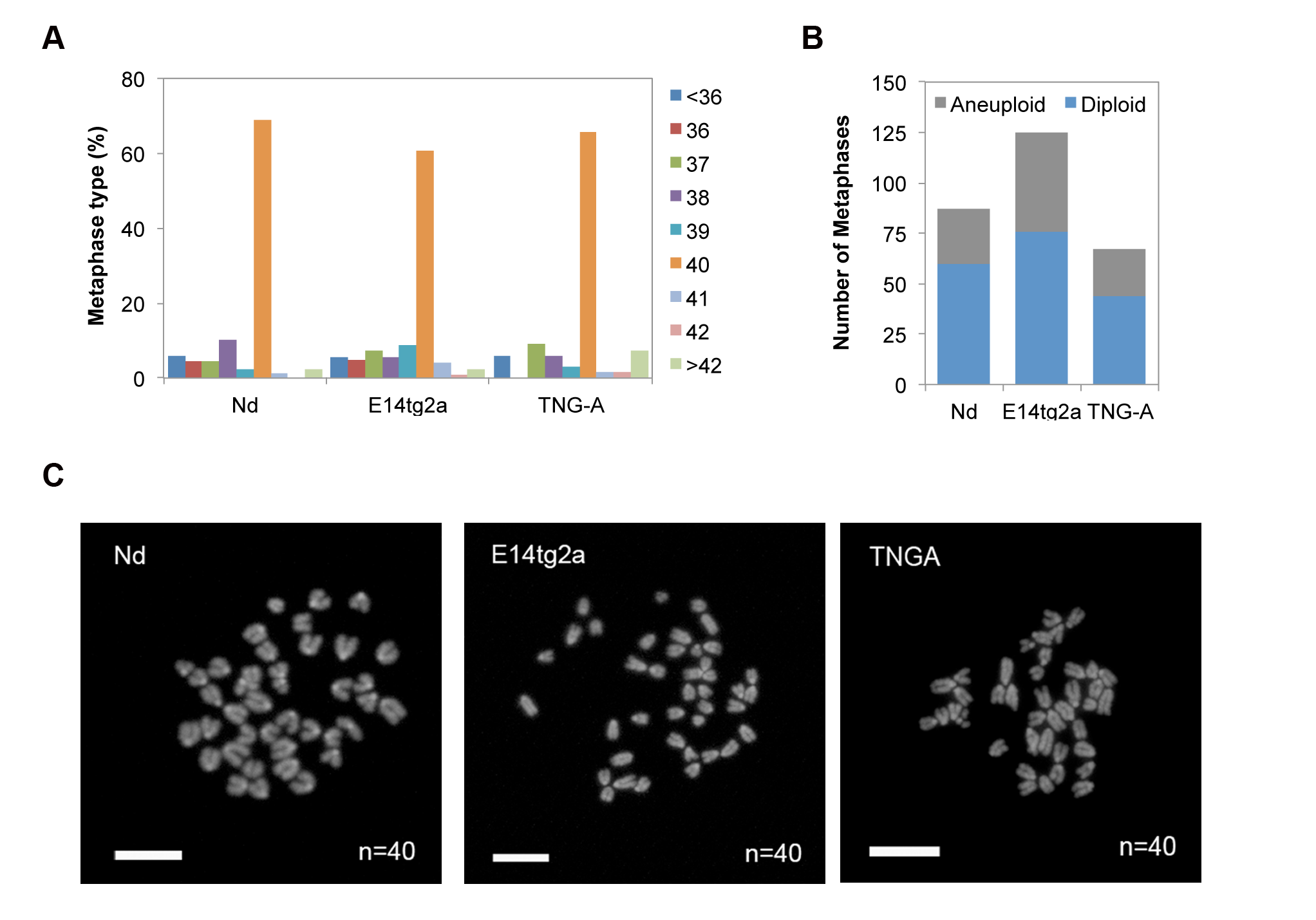

Supplement: Figure S3 — Karyotypic analysis of Nd, E14tg2a and TNG-A ES cell lines. (A) Percentages of diploid (n = 40) and aneuploid metaphases for Nd cells at passage 8, E14tg2a cells at passage 22 and TNG-A cells at passage 40. (B) Total number of metaphases counted. (C) Microphotographs of typical diploid metaphases for each cell line. Scale bar = 10 µm. (TIF) [file pone.0059928.s003.tif]

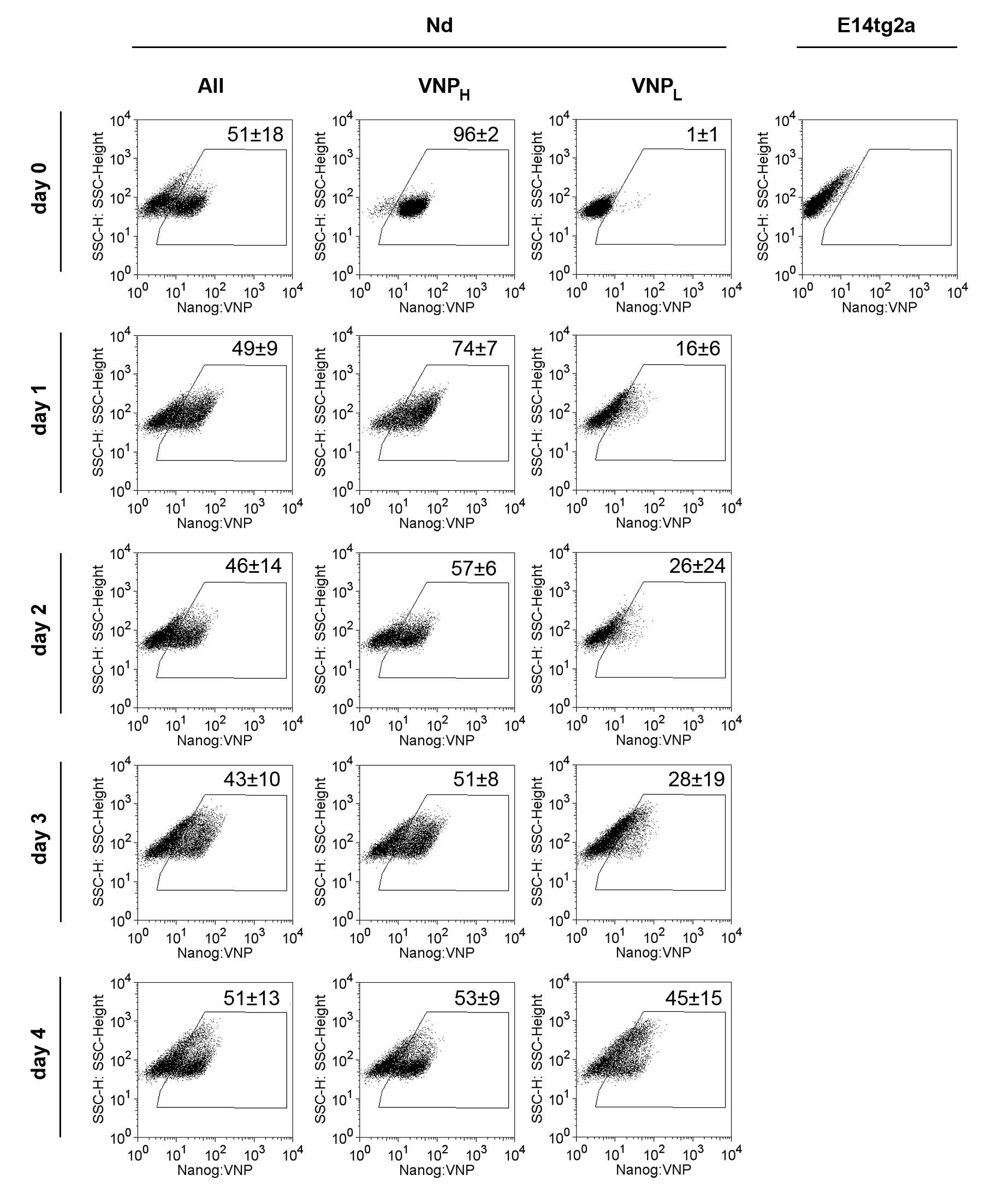

Supplement: Figure S4 — Representative dot blots for Nanog:VNP expression after re-plating of Nd ES cells subpopulations in serum/LIF. FACS sorted populations of Nd ES cells (VNPL and VNPH) were replated in serum/LIF and reporter’s expression was measured for four consecutive days (see Figure 5B). After 2–4 days, heterogeneity is re-established and expression of Nanog:VNP is similar between the three populations, either derived from the sorted VNPL and VNPH subsets, or from the whole population (“All”). The observed rate of reversion is faster and more robust for the VNPH than for the VNPL subpopulations (respectively, 2 and 4 days). E14tg2a cells were used as a negative control, to obtain the positive gate region. (TIF) [file pone.0059928.s004.tif]
