# Supplementary material for: Generation and Characterization of a Novel Mouse Embryonic Stem Cell Line with a Dynamic Reporter of Nanog Expression
Source: PLoS One. 2013 Mar 19;8(3):e59928. doi: 10.1371/journal.pone.0059928 (PMC3602340; doi:10.1371/journal.pone.0059928)
Supplement: Table S1 — List of antibodies used for the immunostaining analyses. (DOCX) [file pone.0059928.s005.docx]

**Table S1.** List of antibodies used for the immunostaining analyses.

| **Antibody anti-…** | **Dilution** | **Animal** | **Origin** |
| --- | --- | --- | --- |
| GFP | 1/500 | Mouse | Abcam #ab1218 |
| GFP | 1/500 | Rabbit | Abcam #ab290 |
| Nanog | 1/150 | Rabbit | Cosmo Bio #RCAB0002P-F |
| Oct4 | 1/500 | Mouse | Santa Cruz #sc-5279 |
| Sox2 | 1/200 | Rabbit | Chemicon #AB5603 |
