# Supplementary material for: Generation and Characterization of a Novel Mouse Embryonic Stem Cell Line with a Dynamic Reporter of Nanog Expression
Source: PLoS One. 2013 Mar 19;8(3):e59928. doi: 10.1371/journal.pone.0059928 (PMC3602340; doi:10.1371/journal.pone.0059928)
Supplement: Table S2 — List of gene-specific primers used in RT-PCR and primers used for BAC recombineering. (DOC) [file pone.0059928.s006.doc]

**Table S2.** List of gene-specific primers used in RT-PCR and primers used for BAC recombineering.

| **Gene** | **Reference** | **T (ºC)** | **Fragment (bp)** | **Forward Primer (5’ – 3’)** | **Reverse Primer (5’ – 3’)** |
| --- | --- | --- | --- | --- | --- |
| **FGF5** | [1] | 60º | 394 | CTTCTGCCTCCTCACCAGTC | CACTCTCGGCCTGTCTTTTC |
| **GAPDH** | [1] | 60º | 466 | ATTCAACGGCACAGTCAAGG | TGGATGCAGGGATGATGTTC |
| **Nanog** | [2] | 57º | 464 | ATGAAGTGCAAGCGGTGGCAGAAA | CCTGGTGGAGTCACAGAGTAGTTC |
| **Oct4** | [1] | 62º | 484 | CTGAGGGCCAGGCAGGAGCACGAG | CTGTAGGGAGGGCTTCGGGCACTT |
| **Sox2** | [2] | 65º | 192 | ATGGACAGCTACGCGCAC | CGAGCCGTTCATGTAGGTCTG |
| **Dusp6** | this paper | 58º | 142 | GTGGTGCTGTACGACGAGAA | CGGCCTGGAACTTACTGAAG |
| **Gata4** | this paper | 60º | 522 | GGCCTCTATCACAAGATGAACG | TCTGCCACTTCTTTCCAATAC |
| **Gata6** | this paper | 58º | 285 | GCCAACTGTCACACCACAAC | TGAGGTGGTCGCTTGTGTAG |
| **Rex1** | this paper | 60º | 233 | AGGCCAGTCCAGAATACCAG | GGAACTCGCTTCCAGAACCT |
| **Primers** | **Reference** | **Sequence (5’ – 3’)** | | | |
| Nanog 5´HA forward | this paper | CG*GGATCC*GCTGAAAGGAAAGCCGTGTA | | | |
| Nanog 5´HA reverse | this paper | CCTCGCCCTTGCTCACCATAGAAAGAAGAGTTAAATGTC | | | |
| Nanog 3´HA forward | this paper | GA*GCGGCCGC*GACTTACGCAACATCTGGGC | | | |
| Nanog 3´HA reverse | this paper | GC*TCTAGA*GCATGTTCTAAGTCCTAGGTTTG | | | |
| VNP polyA forward | this paper | ATGGTGAGCAAGGGCGAGG | | | |
| VNP polyA reverse | this paper | CAG*GTCGAC* GGATCTCCATAAG | | | |

**References:**

1. Abranches E, Silva M, Pradier L, Schulz H, Hummel O, et al. (2009) Neural Differentiation of Embryonic Stem Cells <italic>In Vitro</italic>: A Road Map to Neurogenesis in the Embryo. PLoS ONE 4: e6286.

2. Ying QL, Nichols J, Chambers I, Smith A (2003) BMP induction of Id proteins suppresses differentiation and sustains embryonic stem cell self-renewal in collaboration with STAT3. Cell 115: 281-292.
