# Supplementary material for: Generation and Characterization of a Novel Mouse Embryonic Stem Cell Line with a Dynamic Reporter of Nanog Expression
Source: PLoS One. 2013 Mar 19;8(3):e59928. doi: 10.1371/journal.pone.0059928 (PMC3602340; doi:10.1371/journal.pone.0059928)
Supplement: Table S3 — Protein expression: statistical analysis. All p-values were calculated using a two-tailed distribution, two-sample equal variance t-test. Statistical significant p-values (p-value <0.05) are highlighted in bold, while non-significant differences (p-value >0.05) are depicted in italic. (A) p-values for each cell line grown in serum/LIF vs 2i media. In both culture conditions, similar OCT4 and SOX2 expressions are observed for all cell lines, while statistically significant differences exist in NANOG expression levels. For Nd ES cells, statistically significant differences are additionally observed in VNP expression. (B) p-values for Nd vs E14tg2a cells. In all analysis, no statistically significant difference is observed between Nd and E14tg2a ES cells. (C) p-values for Nd vs TNG-A cells. Statistically significant differences are only observed in VNP/GFP expression when Nd and TNG-A cells are grown in serum/LIF conditions. (DOCX) [file pone.0059928.s007.docx]

**Table S3. Protein expression: statistical analysis.**

All p-values were calculated using a two-tailed distribution, two-sample equal variance t-test. Statistical significant p-values (p-value < 0.05) are highlighted in bold, while non-significant differences (p-value > 0.05) are depicted in italic.

(A) p-values for each cell line grown in serum/LIF vs 2i media. In both culture conditions, similar OCT4 and SOX2 expressions are observed for all cell lines, while statistically significant differences exist in NANOG expression levels. For Nd ES cells, statistically significant differences are additionally observed in VNP expression. (B) p-values for Nd vs E14tg2a cells. In all analysis, no statistically significant difference is observed between Nd and E14tg2a ES cells. (C) p-values for Nd vs TNG-A cells. Statistically significant differences are only observed in VNP/GFP expression when Nd and TNG-A cells are grown in serum/LIF conditions.

| **(A)** | **p-values** (serum/LIF vs 2i**)** | **Protein** | **Nd** | **E14tg2a** | **TNG-A** |
| --- | --- | --- | --- | --- | --- |
|  | **IF** | NANOG | x | x | x |
|  |  | OCT4 | x | x | x |
|  |  | SOX2 | x | x | x |
|  |  | VNP/GFP | x | x | x |
|  | **FC-IS** | NANOG | **0.008** | **0.021** | **0.050** |
|  |  | OCT4 | *0.973* | *0.840* | *0.490* |
|  |  | SOX2 | *0.710* | *0.918* | *0.662* |
|  |  | VNP/GFP | **8.08x10^-7^** | x | *0.536* |
|  | **FC** | *Nanog*:VNP  *Nanog*:GFP | **0.002** | x | *0.369* |
| **(B)** | **p-values**  (Nd vs E14) | **Protein** | **Serum/LIF** | **2i** |  |
|  | **IF** | NANOG | *0.620* | x |  |
|  |  | OCT4 | *0.337* | x |  |
|  |  | SOX2 | *0.739* | x |  |
|  |  | VNP | x | x |  |
|  | **FC-IS** | NANOG | *0.277* | *0.316* |  |
|  |  | OCT4 | *0.472* | *0.464* |  |
|  |  | SOX2 | *0.233* | *0.210* |  |
|  |  | VNP/GFP | x | x |  |
|  | **FC** | *Nanog*:VNP  *Nanog*:GFP | x | x |  |
| **(C)** | **p-values**  (Nd vs TNG-A) | **Protein** | **Serum/LIF** | **2i** |  |
|  | **IF** | NANOG | x | x |  |
|  |  | OCT4 | x | x |  |
|  |  | SOX2 | x | x |  |
|  |  | VNP | x | x |  |
|  | **FC-IS** | NANOG | *0.816* | *0.405* |  |
|  |  | OCT4 | *0.772* | *0.505* |  |
|  |  | SOX2 | *0.850* | *0.772* |  |
|  |  | VNP/GFP | **0.041** | *0.586* |  |
|  | **FC** | Nanog:VNP  Nanog:GFP | **0.012** | *0.789* |  |
