# Supplementary material for: Correction: Generation and Characterization of a Novel Mouse Embryonic Stem Cell Line with a Dynamic Reporter of Nanog Expression
Source: PLoS One. 2014 Jun 20;9(6):e101028. doi: 10.1371/journal.pone.0101028 (PMC4065114; doi:10.1371/journal.pone.0101028)
Supplement: Supplementary file 1 — File S1. Originally published, uncorrected article. (PDF) [file pone.0101028.s001.pdf]

Generation and Characterization of a Novel Mouse Embryonic Stem Cell Line with a Dynamic Reporter of Nanog Expression

Elsa Abranches[1,2]*, Evguenia Bekman[1], Domingos Henrique[1,2]

1 Instituto de Medicina Molecular and Instituto de Histologia e Biologia do Desenvolvimento, Faculdade de Medicina da Universidade de Lisboa, Lisboa, Portugal, 2 Champalimaud Neuroscience Programme, Champalimaud Centre for the Unknown, Doca de Pedroucos, Lisbon, Portugal

Abstract

Background: The pluripotent state in embryonic stem (ES) cells is controlled by a core network of transcription factors that includes Nanog, Oct4 and Sox2. Nanog is required to reach pluripotency during somatic reprogramming and is the only core factor whose overexpression is able to oppose differentiation-promoting signals. Additionally, Nanog expression is known to fluctuate in ES cells, and different levels of Nanog seem to correlate with ES cells' ability to respond to differentiation promoting signals. Elucidating how dynamic Nanog levels are regulated in pluripotent cells and modulate their potential is therefore critical to develop a better understanding of the pluripotent state.

Methodology/Principal Findings: We describe the generation and validation of a mouse ES cell line with a novel Nanog reporter (Nd, from Nanog dynamics), containing a BAC transgene where the short-lived fluorescent protein VNP is placed under Nanog regulation. We show that Nanog and VNP have similar half-lives, and that Nd cells provide an accurate and measurable read-out for the dynamic levels of Nanog. Using this reporter, we could show that ES cells with low Nanog levels indeed have higher degree of priming to differentiation, when compared with high-Nanog cells. However, low-Nanog ES cells maintain high levels of Oct4 and Sox2 and can revert to a state of high-Nanog expression, indicating that they are still within the window of pluripotency. We further show that the observed changes in Nanog levels correlate with ES cell morphology and that Nanog dynamic expression is modulated by the cellular environment.

Conclusions/Significance: The novel reporter ES cell line here described allows an accurate monitoring of Nanog's dynamic expression in the pluripotent state. This reporter will thus be a valuable tool to obtain quantitative measurements of global gene expression in pluripotent ES cells in different states, allowing a detailed molecular mapping of the pluripotency landscape.

Citation: Abranches E, Bekman E, Henrique D (2013) Generation and Characterization of a Novel Mouse Embryonic Stem Cell Line with a Dynamic Reporter of Nanog Expression. PLoS ONE 8(3): e59928. doi:10.1371/journal.pone.0059928

Editor: Qiang Wu, National University of Singapore, Singapore

Received December 2, 2012; **Accepted** February 20, 2013; **Published** March 19, 2013

Copyright: © 2013 Abranches et al. This is an open-access article distributed under the terms of the Creative Commons Attribution License, which permits unrestricted use, distribution, and reproduction in any medium, provided the original author and source are credited.

Funding: This work was funded by "Fundação para a Ciência e a Tecnologia", Portugal (SFRH/BPD/26854/2006 to EA and PTDC/SAU-OBD/100664/2008) (http://alfa.fct.mctes.pt/). The funders had no role in study design, data collection and analysis, decision to publish, or preparation of the manuscript.

Competing Interests: Domingos Henrique is a PLOS ONE Editorial Board member and that this does not alter the authors' adherence to all the PLOS ONE policies on sharing data and materials.

* E-mail: elsa.abranches@gmail.com

Introduction

Embryonic Stem (ES) cells are characterized by their self-renewal capacity and pluripotenciality [1,2]. These cells can be derived from the inner cell mass (ICM) of the mammalian blastocyst and can be maintained in vitro under very specific culture conditions ([3,4], reviewed in [5]). Due to their properties, ES cells constitute a promising resource for the next-generation of cellular therapies; however, scientific, technological and ethical questions are still preventing the development of ES cell-based techniques. One of the major bottlenecks has been the lack of a conceptual understanding of the pluripotent state, which has not emerged yet from the systematic molecular characterization of various pluripotent stem cells.

Recent work has led to a novel view of pluripotency in ES cells as a self-maintaining and intrinsically-controlled "ground state" [6,7], regulated by a gene regulatory network (GRN) in which the transcription factors (TFs) Nanog, Oct4 and Sox2 (NOS network) play a central role [7–9]. Extensive characterization of the transcriptional program elicited by these three TFs revealed that they function in concert to sustain the ES cell state by activating other pluripotency genes while, simultaneously, repressing differentiation-promoting genes [7,9,10]. This repression is thought to play a central role in maintaining the pluripotent state, reducing its vulnerability to the myriad of extrinsic signals that promote differentiation along the various embryonic lineages. However, recent work has shown that both Oct4 and Sox2 can also function as lineage specifiers, assisting the emergence of mesendodermal and neuroectodermal fates, respectively [11,12]. These findings support a different view of the pluripotent state, as a highly unstable and transient cellular state, driven by the competing lineage-promoting activities of the different "pluripotency" factors [13], instead of a ground state implemented and maintained by the NOS circuitry. This scenario emphasizes the precarious and

volatile nature of this state and challenges the idea of an intrinsic ability of ES cells to sustain their state based on a dedicated genetic network. The question therefore remains as to which functions do the pluripotency factors play in establishing and maintaining the pluripotent state of ES cells.

One feature that distinguishes Nanog from its partners Sox2 and Oct4 is the reported heterogeneous expression of this TF in ES cell cultures (and also in the blastocyst's ICM), with some cells showing high levels of Nanog expression while others exhibit reduced levels [14,15]. Furthermore, cells with low or no Nanog expression can evolve into a high-expression state, implying that Nanog levels fluctuate in individual ES cells (contrarily to Oct4 and Sox2) [16,17]. Nanog was initially discovered by virtue of its capacity to oppose differentiation-promoting signals, being essential to maintain ES cells in the absence of LIF/STAT3 signalling [18,19]. This led to the hypothesis that fluctuating levels of Nanog confer different degrees of responsiveness to differentiation signals in individual ES cells, resulting in distinct cellular outputs upon differentiation stimuli [16]. The resulting population heterogeneity might be central to the pluripotent state: on one side, it ensures that there is always a fraction of cells primed to differentiate within a stem cell population, a condition of pluripotency; on the other side, it ensures that other cells are resistant to differentiation cues and keep their pluripotency condition, thereby supporting the self-renewal property.

It is therefore crucial to understand how fluctuations in Nanog levels are generated, and how these fluctuations endow ES cells with various degrees of responsiveness to differentiation cues. To address these questions, a method to monitor in real time the dynamic Nanog expression is required, and we report here the generation of a mouse ES cell line comprising a novel Nanog:VNP reporter (Nd, from Nanog dynamics) that faithfully reflects Nanog expression in ES cells. This reporter provides a powerful tool to dissect the molecular mechanisms regulating Nanog functions in pluripotent cells.

Results and Discussion

Generation of a Nanog:VNP Reporter ES Cell Line

To monitor the dynamic Nanog expression in ES cells, we have generated a novel ES cell line containing a transgenic Bacterial Artificial Chromosome (BAC) with a fluorescent reporter inserted under the control of the *Nanog* regulatory regions. This strategy leaves intact the two endogenous *Nanog* alleles, precluding in this way possible haploinsufficiency phenotypes in ES cells. In addition, since BACs contain large regions of the genome including most, if not all, of the gene-controlling regions, transgene expression is expected to occur in a pattern identical to that of the endogenous gene [20]. A BAC centred around the *Nanog* gene was chosen (clone RP24-464B14) and the *Nanog* coding sequence was replaced with a cDNA encoding the VNP fluorescent protein [21,22], using recombineering technology (Figure 1A) [20]. VNP is a short-lived reporter consisting of a very bright and fast maturating yellow fluorescent protein (Venus), fused to a nuclear localization signal (NLS) and to a degradation signal (PEST). The recombined BAC was electroporated into E14tg2a ES cells, and neomycin selection was applied to select clones with BAC transgene integration. Eight independent VNP-expressing clones were isolated, expanded and characterized. All clones showed similar morphology, viability and growth rate (Figure S1A). However, due to likely differences in the BAC integration site, the percentage of Nanog:VNP-expressing cells was different in each clone, ranging from 4% to 60% as measured by flow cytometry (FC) (Figure S1B).

To select an ES cell clone whose levels of Nanog:VNP transgene expression closely mimic endogenous Nanog expression, we first determined the percentage of Nanog-positive cells in the parental E14tg2a ES cells, as well as in the TNG-A ES cell line (which contains a stable GFP inserted into one of the *Nanog* alleles) [16], grown in standard self-renewal conditions (serum/LIF). This was done by immunofluorescence (IF) in fixed ES cells with an anti-Nanog antibody (Figure 2A), and also by flow cytometry analysis following intracellular staining (FC-IS) with the same antibody (Figure 2B). Our results indicate that, at each given time, approximately 55% of ES cells express Nanog, detected exclusively in the nucleus by IF (Figure 2B, Table 1). We have also quantified the number of Oct4 and Sox2-positive cells in E14tg2a and TNG-A ES cells, and found that more than 90% of cells contain these two proteins in the nucleus (Figure 2C, Table 1).

From the various BAC ES clones (Figures S1B), only the "Nd" clone showed a percentage of Nanog:VNP positive cells (57±13%, Figure S1B) similar to that of Nanog-positive cells in E14tg2a and TNG-A cell lines. This clone was thus selected for further characterization. DNA analysis of this clone shows that only one copy of the BAC was integrated (Figure S2), and mRNA analysis by Northern blot reveals that the transcript encoding the VNP reporter has the expected size, indicating that BAC integration did not disrupt the reporter cassette. In addition, the levels of *VNP* and *Nanog* mRNAs are regulated in parallel, decreasing after LIF removal and increasing after culture in ground state (2i) conditions (Figure 1B), showing that reporter expression is properly controlled by the upstream *Nanog* regulatory regions.

We next analysed the percentage of cells positive for Sox2, Oct4 and endogenous Nanog in the selected Nd clone. The values were very similar to those obtained for both E14tg2a and TNG-A cells (Figure 2B,C, Table 1), showing that the integration of the Nanog:VNP BAC does not interfere with the normal expression of these pluripotency genes. More importantly, both double-IF analysis (Figure 2A) and FC-IS analysis (Figure 2C) reveal a strong correlation between Nanog and VNP expression in Nd ES cells (Figure 2D), indicating that the Nanog:VNP reporter provides an accurate fluorescent read-out for Nanog expression in ES cells. In contrast, TNG-A ES cells show more GFP-expressing cells than Nanog-positive cells (81.7±1.1% compared with 54.8±13.2%, Figure 2B,E, Table 1), a result most likely due to perdurance of the stable GFP protein used as a reporter in this ES cell line.

To further assess the adequacy of the VNP reporter, we measured the protein half-life times for VNP, Nanog, Oct4 and GFP in the various ES cell lines (Figure 3A): while GFP is stable for at least 6 hours in TNG-A cells, without any decay after translational inhibition, both Nanog and VNP have similar half-lives (2.3 hours and 1.8 hours, respectively), justifying the use of VNP as an adequate reporter for Nanog expression. As expected, Oct4 is stable during the studied period of time, for all the ES cell lines.

Lastly, we have measured the *VNP*, *Nanog*, *Oct4* and *Sox2* mRNAs' half-lives, in E14tg2a and Nd cell lines (Figure 3B). While *Oct4* mRNA is stable in both cell lines, all other transcripts show fast decay rates, with *Sox2* mRNA being the more short-lived, while *VNP* and *Nanog* mRNAs have very similar half-lives (~4hours).

Growth and Differentiation of Nd ES Cells are Similar to the Parental Cell Line (E14tg2a)

To further validate the Nd ES cell line, we compared its growth characteristics with the parental E14tg2a cell line, in serum/LIF conditions. Both ES cell lines grew as typical ES colonies

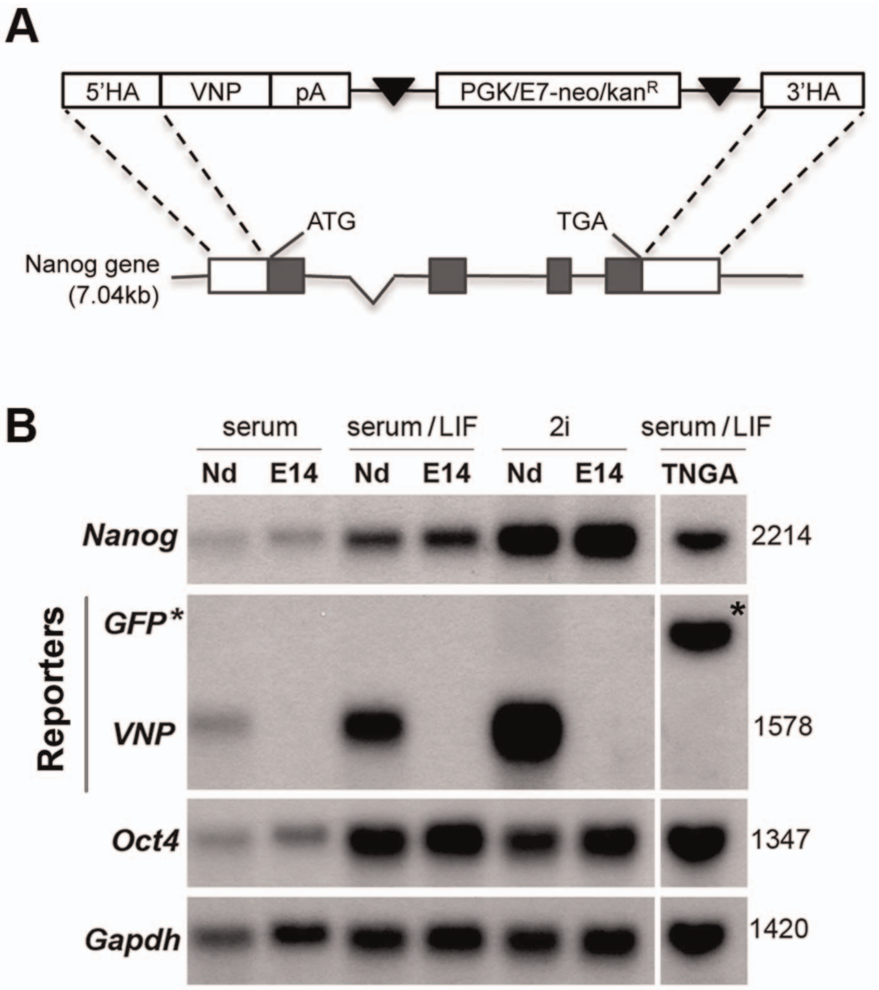

Figure 1. Nanog:VNP reporter cassette and Northern blot analysis of E14tg2a, TNG-A and Nd ES cell lines. (A) Scheme of the recombination cassette containing the VNP cDNA and a neomycin resistance gene, inserted into the BAC (clone RP24-464B14) downstream of the *Nanog* starting codon. (B) Northern blot analysis of E14tg2a (E14) Nd and TNG-A ES cells probed for Nanog, Oct4, Gapdh (housekeeping gene) and reporter mRNAs, in self-renewal (serum/LIF and 2i) and differentiation (serum) conditions. Transcript sizes are indicated on the right. Higher levels of Nanog RNA are detected for both E14 and Nd ES cells in 2i conditions, compared to serum/LIF, while Oct4 is highly expressed in both cases. VNP mRNA is only detected in Nd ES cells. In differentiation conditions (serum alone), Nanog, Oct4 and VNP mRNA expression is strongly reduced. GFP* refers to the fusion transcript present in TNG-A ES cells, arising from the recombination of a GFP cDNA into the Nanog locus [16]. This fusion transcript has a similar size to the endogenous *Nanog* mRNA expressed from the other allele.
doi:10.1371/journal.pone.0059928.g001

throughout several passages (n≥8), with similar proliferation rates (shown as fold increase in Figure 4A) and viability (above 95%, Figure 4A). In addition, both ES lines maintained a normal diploid karyotype (Figure S3).

We also assessed the differentiation potential of the Nd cell line, using two different methods: formation of embryoid bodies (EBs) and monolayer neural differentiation [23]. In both conditions, Nd cells show normal differentiation patterns, generating EBs composed of cells from the three germ layers, and forming typical neuroepithelial rosette-like structures when grown in monolayer conditions in the absence of LIF (Figure 4B). Comparing RT-PCR results obtained for ES cells and day 4 EBs, we observed the expected downregulation of known pluripotency genes (*Nanog*, *Oct4*, *Sox2* and *Rex1*) along differentiation, concomitantly with up-regulation of ectoderm (*Fgf5*), mesoderm (*T-brachyury*) and endoderm (*Gata6*) lineage-markers (Figure 4C). Overall, these data confirm the pluripotent characteristics of the Nd cell line.

Monitoring Nanog Heterogeneity and Expression Dynamics with Nd ES Cells

Previous work indicates that Nanog expression in ES cells is highly dynamic, with fluctuating levels in individual cells [16,24], resulting in the observed heterogeneous distribution of Nanog-high and Nanog-low cells in populations of ES cells grown in serum/LIF conditions. To test whether the Nd reporter cell line is adequate to monitor these reversible alterations in the levels of Nanog expression, we followed the growth of FACS-purified subsets of Nd cells with either low VNP (VNP_L) or high VNP (VNP_H) levels (Figure 5A), in identical self-renewal conditions (serum/LIF). Samples were taken every 24 hours, for 4 days, and analysed for the distribution of Nanog:VNP expressing cells. The results (Figure 5B, Figure S4) show that both subsets of Nd ES cells can re-establish the normal heterogeneous distribution of VNP levels after 2–4 days in culture. These results are comparable to those reported for TNG-A ES cells containing a stable GFP knocked-in the *Nanog* locus [16], although the restoration of the

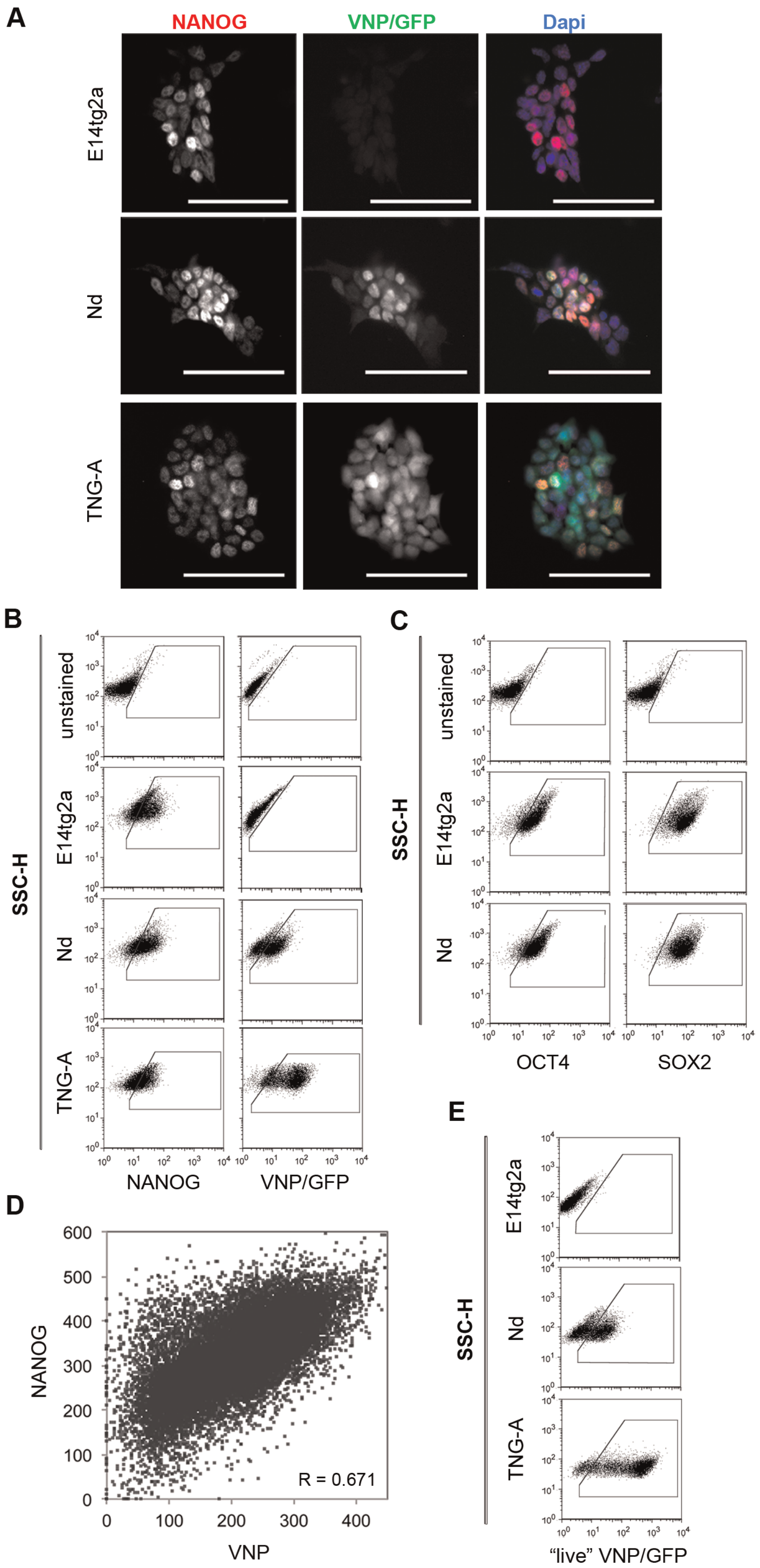
A
NANOG
VNP/GFP
Dapi
E14tg2a
Nd
TNG-A
B
SSC-H
unstained
E14tg2a
Nd
TNG-A
NANOG
VNP/GFP
C
SSC-H
unstained
E14tg2a
Nd
OCT4
SOX2
D
NANOG
VNP
R = 0.671
E
SSC-H
E14tg2a
Nd
TNG-A
"live" VNP/GFP

Figure 2. NOS network and reporters expression for cell lines grown in serum/LIF conditions. (A) Immunofluorescence detection for Nanog (red) and VNP/GFP (green) in E14tg2a, Nd and TNG-A cell lines; nuclei counterstained with DAPI (blue). Nanog expression is observed in approximately 50% of cells for all cell lines, while VNP is expressed in the same percentage of Nd cells, with a high degree of co-localization. Higher number of GFP positive cells than Nanog-positive is observed for TNG-A cells. Scale-bar = 50 um. (B) Representative dot blots for FC-IS analysis of Nanog and VNP/GFP for E14tg2a, Nd and TNG-A cells. Negative controls (samples with no primary antibody) for both Nanog and VNP/GFP staining's are shown, based on which the positive gate regions were designed. Nanog expression is similar between different cell lines grown in the same media. In Nd cells, VNP levels mimic Nanog expression in both culture conditions, while in TNG-A cells, GFP levels are much higher than Nanog levels, most likely due to reporter stability. (C) Representative dot blots for FC-IS analysis of Oct4 and Sox2 for E14tg2a and Nd. Negative controls (samples with no primary antibody) for both Oct4 and Sox2 staining's are shown, based on which the positive gate regions were designed. Oct4 and Sox2 levels are always expressed in more than 90% of the cells in both media conditions. (D) Correlation plot for Nanog and VNP proteins expression in Nd cells. Graph depicts data from 24906 cells from three biological replicates obtained by FC-IS. Statistical analysis indicates a high degree of correlation between the expression of both proteins (Pearson correlation = 0.647). (E) Representative dot blots for FC analysis of live VNP/GFP for E14tg2a, Nd and TNG-A cells grown. E14tg2a cells were used as negative controls, based on which positive gate regions were designed. Obtained data is similar to that obtained by FC-IS (C). Quantifications of (A–C,E) may be observed in Table 1.
doi:10.1371/journal.pone.0059928.g002

original distribution was considerably faster and more complete in Nanog:VNP Nd ES cells. We have also analysed by RT-PCR the expression of various pluripotency and lineage-specific markers in the initial sorted populations, as well as in the populations formed after 4 days in culture. The results (Figure 5C) indicate that the initial VNP_H and VNP_L subsets have similar levels of *Oct4* and *Sox2* mRNA expression, with an expected lower expression of *Nanog* in the VNP_L subset. However, the expression of lineage markers (*Fgf5*, *Gata6* and *T-brachyury*) is strikingly up-regulated in the sorted VNP_L subset, implying that these cells display a higher degree of priming to differentiation. After 4 days in culture of this VNP_L subset, the expression of these lineage markers seems to reach even higher levels, but this is most likely due to the relatively high number of VNP_L cells still present in 4-day cultures (note the lower VNP expression in lane 6), as a result of the slower rate of restoration of Nanog heterogeneity in these cells, when compared with VNP_H–derived cells (Figure 5B).

Overall, these results indicate that the Nanog:VNP reporter provides a good read-out for the dynamic levels of Nanog expression in ES cells, allowing us to correlate these levels with the variations in differentiation potential that ES cells are constantly experiencing. In addition, our experiments reveal that although VNP_L cells show higher levels of priming to differentiation, they remain within the ground state of pluripotency [7]. This is shown by the persistent high levels of *Oct4* and *Sox2* expression, and by the capacity of VNP_L cells to revert to a state of high *Nanog* expression, re-establishing the typical expression profile of ES cells in self-renewal conditions.

Nanog Levels in ES Cells Grown in "Ground State" Conditions

Maintenance of the pluripotent state in ES cells is known to be dependent on inhibition of differentiation stimuli encoded by FGF/ERK signalling [25,26]. This finding was explored to define improved culture conditions for ES cells, which can be efficiently maintained, without serum, in the presence of highly specific FGF/ERK inhibitors, together with GSK3ß inhibitors (2i media) [6]. To test the behaviour of the Nanog:VNP reporter in these conditions, we compared the growth of Nd cells in serum/LIF versus 2i, to which we added LIF to get optimal culture conditions. FC analysis reveals that 56.2±8.0% of Nd cells show VNP expression in presence of serum/LIF (Figure 2E), and that this percentage increases to 91.1±3.1% (Figure 6A,B, Table1) when Nd cells are grown for 48 hours in 2i. This increase is maintained

Table 1. Protein expression analysis.

Technique	Protein	Nd		E14tg2a		TNG-A	
		serum/LIF	2i	serum/LIF	2i	serum/LIF	2i
IF	NANOG	49.9±3.5	n.d.	51.0±3.8	n.d.	n.d.	n.d.
	OCT4	98.5±1.3	n.d.	97.5±1.4	n.d.	n.d.	n.d.
	SOX2	95.8±2.4	n.d.	96.4±2.3	n.d.	n.d.	n.d.
	VNP/GFP	44.5±3.3	n.d.	residual	n.d.	n.d.	n.d.
FC-IS	NANOG	51.9±11.9	89.2±5.0	57.0±6.7	85.5±11.6	54.8±13.2	92.9±1.6
	OCT4	91.7±5.6	90.2±10.1	91.2±8.3	90.9±7.8	89.5±10.7	96.0±2.8
	SOX2	97.7±2.1	98.3±1.6	96.1±2.6	95.8±4.6	97.2±3.1	98.4±0.8
	VNP/GFP	52.9±6.4	84.6±3.3	residual	residual	81.7±1.1	84.5±5.3
FC	*Nanog*:VNP *Nanog*:GFP	56.2±8.0	91.1±3.1	residual	residual	88.9±1.4	92.0±3.5

n.d. = not determined.
Expression of Nanog, Oct4, Sox2, VNP and GFP proteins in Nd, E14tg2a and TNG-A cells, grown in serum/LIF or 2i media, and measured by IF, FC-IS or FC in non-fixed cells. Averages and standard deviations of at least 3 independent experiments are depicted (except for TNG-A cells, where only two biological replicates were performed). Nanog expression is not statistically different between different cell lines grown in the same media, although IF measurements provide slightly lower values than FC-IS, probably due to higher sensitivity of cytometry analysis. ES cells grown in 2i media significantly increase Nanog expression (determined by FC-IS) when compared to serum/LIF conditions for all cell lines. In Nd cells, VNP mimics Nanog expression in both culture conditions. In contrast, the percentage of GFP-positive cells in TNG-A ES cells is much higher than the percentage of Nanog-positive cells in serum/LIF conditions, most likely due to reporter stability. Oct4 and Sox2 are always detected in more than 90% of cells in both media and for all ES cell lines, with no statistically significant differences being observed. See Table S3 for statistical data analysis (p-values).
doi:10.1371/journal.pone.0059928.t001

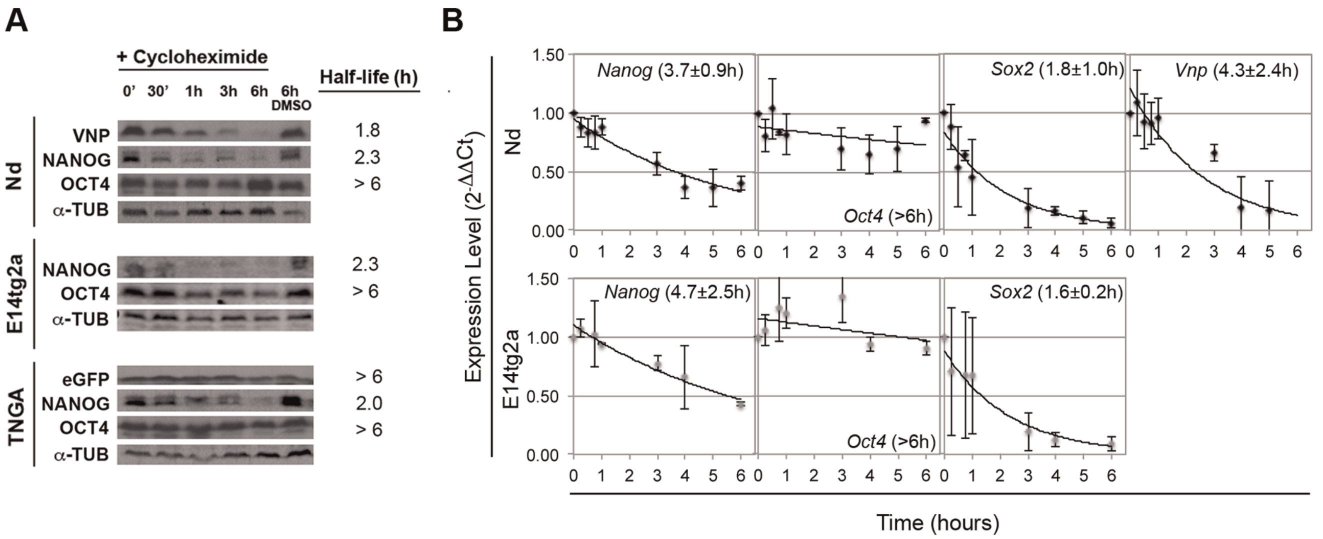

Figure 3. Protein and RNA half-lives of different pluripotency and reporter genes. (A) Western blot analysis for VNP/GFP, Nanog, Oct4and a-Tub proteins in Nd, E14tg2a and TNG-A ES cell lines, over 6 hours after protein synthesis inhibition by cycloheximide (6h DMSO corresponds to the control without cycloheximide addition). Column on the right indicates the decay times of the targeted proteins. (B) Representative quantitative RT-PCR graphs for Vnp, Nanog, Oct4 and Sox2 mRNAs, calculated over 6 hours after transcription inhibition with actinomycin D (averages and standard deviations of at least 2 independent experiments are shown). Half-lives calculated using these data are depicted on each graph in brackets. No statistically significant differences were observed between Nd and E14tg2a cell lines (p-values >0.15).
doi:10.1371/journal.pone.0059928.g003

through three serial passages in 2i media (Figure 6C) and seems to be accompanied by slightly lower, but not statistically different, proliferation rates (data not shown).

We next assessed the expression of the Oct4, Sox2 and Nanog proteins in Nd and E14tg2a cells grown for 48 hours in serum/LIF or 2i. Analysis by FC-IS shows an increase in Nanog-expressing cells in both ES lines (as well as in TNG-A cells), confirming the results obtained with the Nanog:VNP reporter in Nd cells (Figure 2B,C versus Figure 6D,E, Table 1). As expected, the percentage of Oct4 and Sox2 positive cells is similar in both culture conditions. These results were replicated at the mRNA level, as quantitative PCR analysis reveals a statistically significant increase in the expression levels of *VNP* and *Nanog* mRNAs when ES cells are grown in 2i, while *Oct4* and *Sox2* expression does not vary significantly (Figure 6F). Concomitantly, a clear decrease was observed in the expression of *Dusp6*, a known FGF/ERK target [27,28], and of *Fgf5*, a marker for epiblast progression [29]. Together, these results reveal an effective increase in the number of ES cells expressing high levels of Nanog (and of the reporter Nanog:VNP) when FGF/ERK signalling is blocked. Nonetheless, a small population of ES cells with no or low levels of Nanog can be consistently detected in 2i cultures (Figure 6B, asterisk), suggesting that although population heterogeneity is reduced, fluctuations in Nanog expression may still occur in the absence of FGF/ERK signalling.

Nanog Levels Correlate with ES Cell Colony Morphology

ES cell cultures in serum/LIF and 2i media reveal considerably different morphologies (Figure 6G), with ES cell colonies showing a more tightly packed morphology in 2i conditions, and a concomitant reduction in flattened differentiated-like cells at the colonies' periphery. When we reduced the concentrations of FGF/ERK and GSK3ß inhibitors present in 2i media, we could observe that ES cells colonies become less tightly packed and more adherent to the gelatin substrate (Figure 7A). These morphological alterations correlate strikingly with the reduction of Nanog-expressing cells in the whole population (monitored by the Nanog:VNP reporter, Figure 7B,C), suggesting a possible role of Nanog in coordinating the differential expression of cell adhesion molecules in ES cells, as part of its function in modulating the pluripotency state. Actually, it has been shown that spontaneous differentiation of ES cells is accompanied by a decrease in the presence of E-cadherin at the cell surface, probably as part of the epithelial-mesenchymal transition (EMT) necessary for entry into differentiation [30]. Moreover, it has been suggested that EMT inhibition in human ES cells favors the induction of the ground state pluripotency [31], and that Nanog represses expression of Eomes, preventing it from cooperating with SMAD2/3 to activate the transcriptional network directing EMT [32]. Thus, the different cell adhesion behaviour of high-Nanog ES cells might be important to inhibit EMT and to resist entry into differentiation. In addition, this possible role of Nanog in controlling cell adhesion properties might be very important in vivo, for instance during the segregation of epiblast and endoderm lineages in the ICM, where differential profiles of cell adhesion molecules in high-Nanog epiblast progenitors and low-Nanog endoderm progenitors might underlie the cell sorting process that correctly positions the two lineages in the blastocyst [33,34].

Conclusions

In this paper, we describe a novel mouse ES cell line, engineered to have a short-lived fluorescent protein (VNP) as a reporter of Nanog expression. This reporter provides an accurate read-out of Nanog expression, and was used to monitor the behaviour of purified sub-populations of ES cells in different Nanog expression states. Our data confirms that low-Nanog cells have a higher degree of priming to differentiation, while ES cells with high Nanog are in a pristine pluripotent state. Using this novel Nanog reporter, we could observe heterogeneity of Nanog expression even when ES cells are cultured in ground state

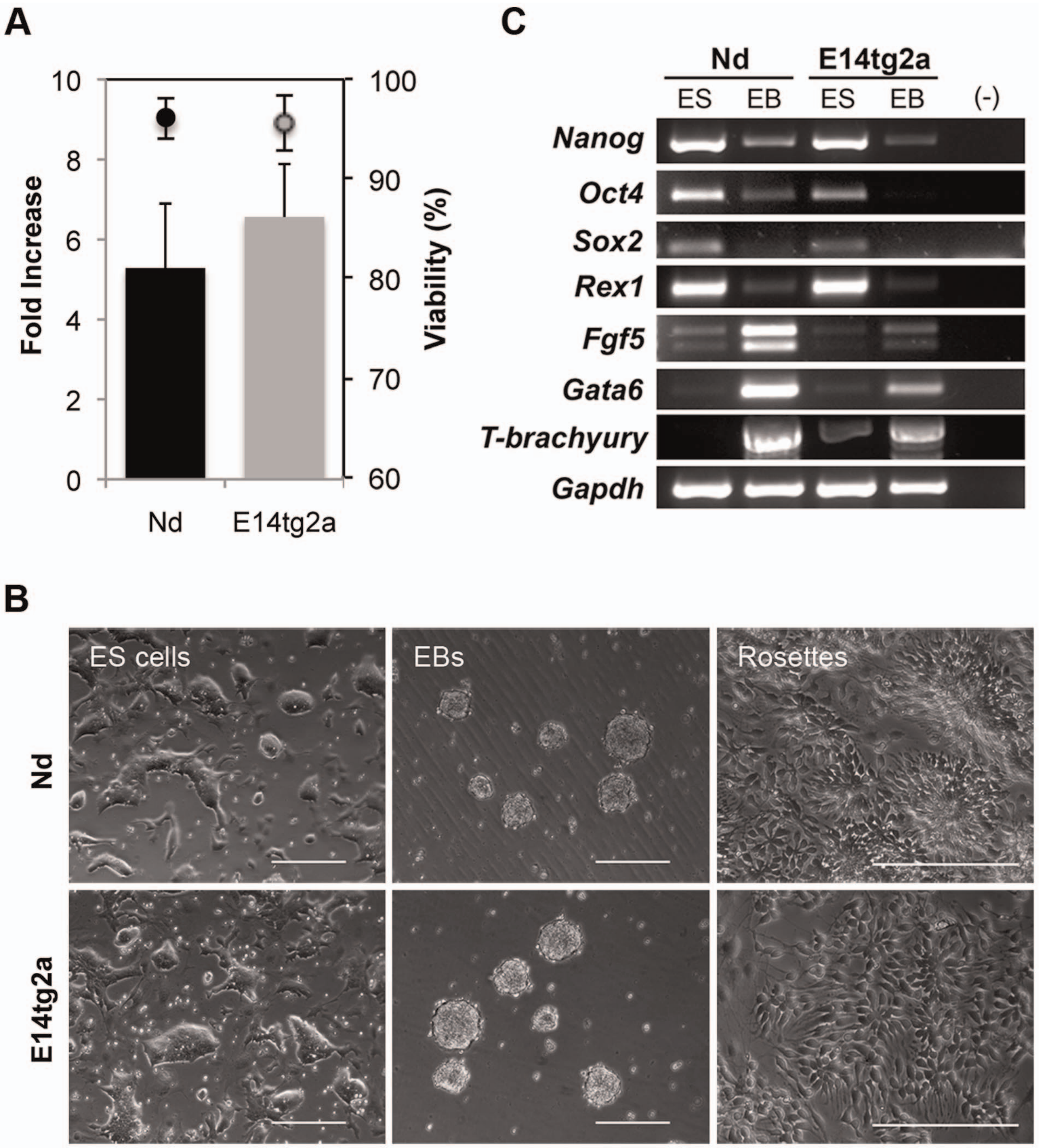

Figure 4. Pluripotency potential of the Nd ES cell line. (A) Cell growth (bars), measured as fold increase, and viability (circles) for Nd and E14tg2a cell lines passaged every 48 h in serum/LIF conditions (n≥8). No statistically significant differences were observed between Nd and E14tg2a cell lines (p-values >0.09). (B) Bright field images of Nd and E14tg2a ES cells in grown in serum/LIF (ES cells growing in clusters), after 4 days of differentiation through EBs formation (cells growing as suspension aggregates), and after 8 days of monolayer neural differentiation (with formation of neural rosettes). Scale-bar = 200 um. (C) RT-PCR analysis of Nd and E14tg2a ES cells and corresponding day 4 EBs for known ES cell markers (*Nanog*, *Oct4*, *Sox2* and *Rex1*) and ecto- (*Fgf5*), meso- (*T-brachyury*) and endoderm (*Gata6*) markers. Upon differentiation, pluripotency genes expression is downregulated while expression of markers from the three germ layers increase.
doi:10.1371/journal.pone.0059928.g004

conditions (2i), in contrast to what has been previously suggested [35].

Finally, we observed that the levels of Nanog expression correlate with different ES cell morphologies, suggesting that Nanog might have a role in coordinating the differential expression of cell adhesion molecules in ES cells.

This novel Nanog reporter ES cell line will therefore be a powerful tool to perform a quantitative and systematic analysis of the pluripotent state, and to dissect the molecular mechanisms that regulate Nanog function.

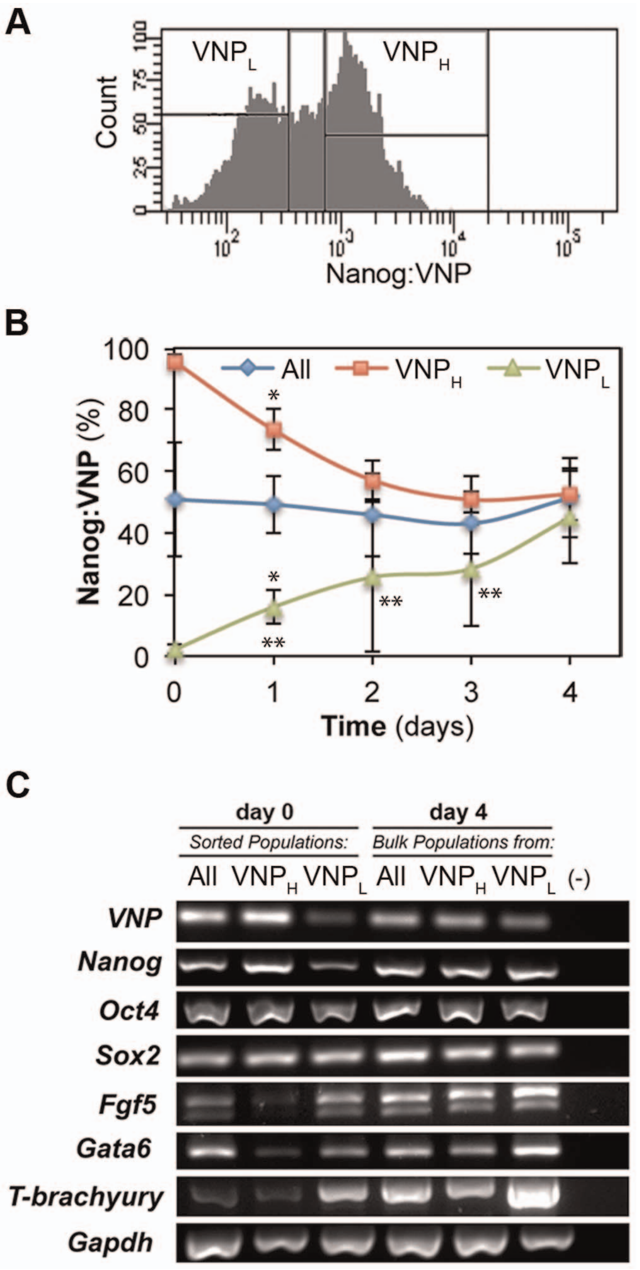

Figure 5. *Nanog* expression in FACS-sorted Nd ES cells. (A) Representative histogram of FACS-sorted Nd sub-populations, grown in serum/LIF. VNP-low (VNP_L) and VNP-high (VNP_H) populations were collected for posterior analysis. (B) Nanog:VNP expression after re-plating sorted populations of Nd ES cells in serum/LIF. After 2–4 days, normal levels of heterogeneity are re-established, and expression of Nanog:VNP is similar between the three populations, either derived from the sorted VNP_L and VNP_H subsets, or from the whole population ("All"). Representative dot blots for FC analysis of VNP in non-fixed cells are presented in Figure S4. Statistically significant differences (p-value <0.05) observed between "All" and VNP sub-populations are denoted with (*), while statistically significant differences between VNP_L and VNP_H are denoted with (**). (C) mRNA expression analysis by RT-PCR of cells collected immediately after sorting (day 0) and four days after re-plating (day 4). The purified VNP_L subpopulation (immediately after sorting) shows lower Nanog mRNA levels and higher expression of lineage-affiliated genes (Fgf5, Gata6 and T-brachyury). After culture for 4 days, both VNP_L and VNP_H subsets show similar Nanog mRNA expression. Expression of lineage markers in the VNP_L subpopulation is still more elevated after 4 days of culture, most likely reflecting the slower reversion to heterogeneity (Figure S4). Similar levels of Oct4 and Sox2 expression are observed for all analysed samples.
doi:10.1371/journal.pone.0059928.g005

Materials and Methods

Maintenance and Differentiation of Mouse ES Cells

The ES cell lines used for this study were E14tg2a and TNG-A (both a kind gift from Austin Smith's lab, University of Cambridge, UK) [16] and Nd, a novel transgenic ES cell line. TNG-A ES cells were derived from E14tg2a ES cells and contain a stable GFP reporter fused to the puromycin resistance gene inserted into the *Nanog* locus [16]. The Nd cell line is a BAC-transgenic ES cell line for VNP-tagged *Nanog* gene, derived from E14tg2a ES cells (see below for BAC-transgenic ES cell line generation).

ES cells were routinely grown at 37°C in a 5% (v/v) CO2 incubator in Glasgow Modified Eagles Medium (GMEM, Invitrogen), supplemented with 10% (v/v) fetal bovine serum (FBS) (ES-qualified, Invitrogen), 2 ng/ml LIF and 1 mM 2-mercaptoethanol (serum/LIF conditions), on gelatin-coated (0.1% (v/v)) dishes (Nunc). Cells were passaged every other day, at constant plating density of 3×10^4 cells/cm^2. Alternatively, ES cells were grown in 2i medium (*iStem* medium, Stem Cells Inc.) [6], supplemented with LIF, a serum-free based medium that contains inhibitors of the ERK pathway and of GSK3ß (2i conditions).

For the EB formation assay, ES cells were seeded on 60-mm bacterial-grade petri dishes at 3×10^4 cells/cm^2, in GMEM medium in the absence of LIF. EBs formed within 24 hours and medium was changed every 2 days. Aggregates growth was monitored under an inverted microscope for 8 days, RNA was collected at day 0 and day 4.

For the neural differentiation assay, the monolayer protocol described in Abranches et al, 2009 was used [23]. Briefly, ES cells were plated at specific cell densities and grown for 4 days in RHB-A medium (Stem Cells Inc.) on gelatin-coated plates. On day 4 cells were dissociated and re-plated onto laminin-coated tissue culture plastic in RHB-A medium supplemented with 5 ng/ml murine bFGF (Peprotech). Medium was changed every 2 days and cells were monitored under an inverted microscope for 8 days for the appearance of neural rosettes.

BAC-transgenic ES Cell Lines Generation

BAC clone RP24-464B14 bearing 213-kb insert containing the *Nanog* gene was ordered from CHORI database (http://bacpac.chori.org). The 5′HA for *Nanog* was PCR amplified from BAC DNA using forward primer 5′- CGGGATCCGCTGAAAG-GAAAGCCGTGTA-3′ and reverse primer 5′-CCTCGCCCTTGCTCACCATAGAAAGAAGAGT-TAAATGTC-3′. VNP sequence followed by rabbit beta-globin polyA was amplified from VNP-pCAGGS vector [22] using forward primer 5′-ATGGTGAGCAAGGGCGAGG-3′ and reverse primer 5′-CAGGTCGAC GGATCTCCATAAG-3′. Both amplification products were joined together by PCR ligation using Phusion DNA Polymerase (Finnzymes), and the resulting 1.9-kb PCR fragment was cloned into pBlueScript KS (Stratagene) and sequenced. The 3′HA for *Nanog* was PCR-amplified from BAC DNA using forward primer 5′-GAGCGGCCGCGACTTACG-CAACATCTGGGC-3′ and reverse primer 5′-GCTCTAGAG-CATGTTCTAAGTCCTAGGTTTG-3′, and cloned downstream of floxed PGK-E7-neoR cassette from PL451 vector (Pentao Liu, Addgene) between NotI and XbaI sites. 5′- and 3′-parts of the final NanogVNP-neoR cassette were joined together by 3-way ligation of BamHI-SalI and SalI-XbaI DNA fragments into pBlueScritp KS digested BamHI-XbaI and end-sequenced. *Nanog* BAC clone RP24-464B14 was transfected with defective

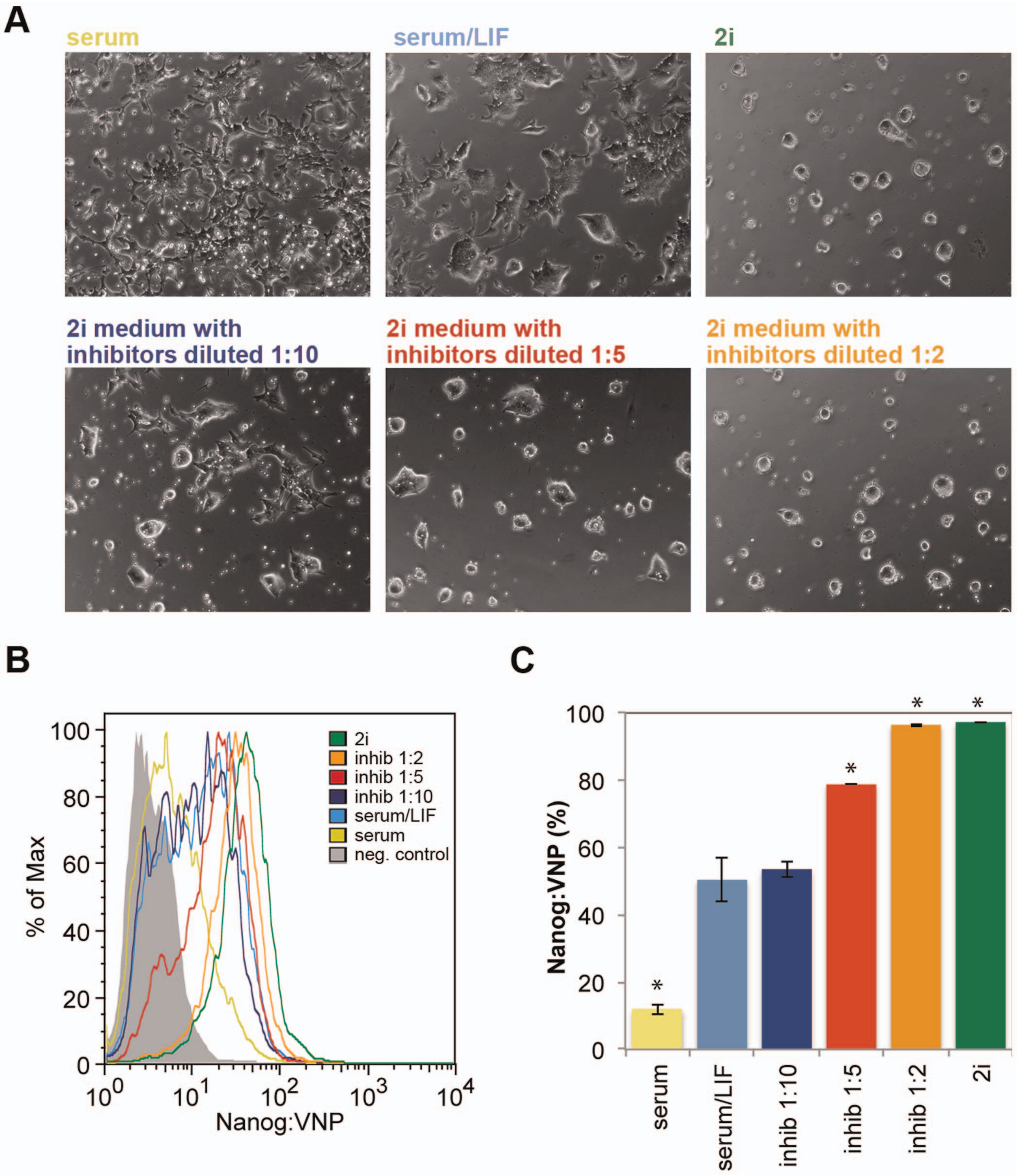

Figure 7. Morphology and Nanog:VNP levels correlation. (A) Bright field images of Nd cells grown in different culture media: serum; serum/LIF; 2i; and 2i with reduced inhibitors (FGF/ERK and GSK3ß inhibitors) concentration (1:10, 1:5 and 1:2). The addition of increasing amounts of inhibitors results in more tightly packed morphology of ES cell colonies and in the reduction in flattened differentiated cells and a more tightly packed morphology of ES cell colonies. When ES cells are grown in serum alone, the inverse is observed. (B) Representative FC histograms of Nanog:VNP expression for Nd cells grown in different culture media.The addition of increasing amounts of inhibitors results in the increasing expression of Nanog:VNP (to up 95%), while cell grown in serum alone decreases significantly Nanog:VNP expression (to around 15%). The negative control cells (E14tg2a) are represented in gray. (C) Nanog:VNP expression quantifications for Nd cells grown in different culture media (n = 3). Statistically significant differences (p-value <0.002) observed between ''serum/LIF'' and other conditions are denoted with (*).
doi:10.1371/journal.pone.0059928.g007

lambda prophage bearing RET-ET recombineering system under thermolabile repressor [36]. Resulting Tet-resistant bacteria were subjected to first recombineering with PISCE plasmid to remove the LoxP site in the backbone of pTARBAC1 vector (reviewed in [20]), and to second recombineering with Nanog-VNP cassette. Tetracycline, ampicillin and neomycin-resistant recombinant BAC clones were selected by PCR screening and pulse-field gel electrophoresis of analytical digests with XhoI, PI-SceI, AscI and SnaBI enzymes. Final recombinant BAC clone was digested with AscI, ethanol precipitated and microdialysed on 0.01 mm filter (Millipore) prior to electroporation in ES cells. The linearized BAC was electroporated into E14tg2a cells (electroporation conditions: 250 V and 500 μF). Electroporated cells were plated in serum/LIF conditions and incubated at 37°C, 5% CO2. After 24 h, neomycin selection was started (200 ug/mL G418) and individual clones were picked after 12 days. Clones were expanded and analysed.

To determine the number of BAC copies integrated in the Nd ES cell line, standard curves for the relative quantities of BAC (pBAC) and of Nanog (pNanog) were determined using a dilution series of DNA plasmids containing each sequence. Threshold cycle (Ct) values were plotted against log-transformed concentrations of plasmid DNA, trend lines were calculated and quantities of pBAC relative to pNanog were determined for the wild-type E14tg2a ES cell line and for the Nd ES cell clone.

Immunocytochemistry

Fixed cells (15 min, 4%PFA) were blocked with 10% (v/v) FBS and 0.05% (v/v) Tween in phosphate buffered saline (PBS) for 1 hour, followed by overnight incubation with primary antibodies (Table S1). Cells were washed 3 times in PBS followed by incubation for 1–2 hours with AlexaFluor-conjugated secondary antibodies (Molecular Probes) and DAPI nuclear stain (1:10000, Sigma). Images of fixed cells were obtained with a DM5000B microscope and a DC350F camera (Leica Wetzlar, Germany). Living cells were photographed under an inverted microscope Leica DMIL with a DC200 camera. Images were processed using Photoshop CS (Adobe, San Jose, CA).

The number of Nanog, GFP/VNP, Oct4 and Sox2 expressing cells was quantified as a proportion of the total number of cells in culture, counted with the ImageJ Cell Counter software. The number of positively labelled cells was quantified by counting 5 to 10 randomly selected fields per coverslip, corresponding to a minimum 1000 cells, counted as DAPI nuclei. Two coverslips were counted per each condition and the analysis was repeated for at least three independent experiments for E14tg2a and Nd ES cell lines.

For FC-IS analysis, 2 ug of each antibody was used per 10^6 cells. Briefly, fixed cells were blocked for 1 hour with 0.25% (w/v) saponin and 5% (v/v) sheep serum in PBS, followed by incubation for 1 hour with primary antibodies. Cells were washed 2 times in 0.25% (w/v) saponin in PBS followed by incubation for 1 hour with AlexaFluor-conjugated secondary antibodies (Molecular Probes).

Flow Cytometry (FC) Analysis

For live cells FC analysis and sorting experiments, cells were dissociated and resuspended in 4% (v/v) FBS in PBS. Nanog:GFP and Nanog:VNP analysis was performed on a FACS Calibur cytometer (Becton Dickinson) and cell sorting experiments were done on a FACS Aria cell sorter (Becton Dickinson). Live cells were gated based on forward scatter and side scatter and/or by propidium iodide dye exclusion. For sorting, VNP positive and VNP negative Nd ES cell populations were collected and cell viability at the end of the FACS sorting procedure was determined using trypan blue dye exclusion method. FACS sorted cells were processed for RNA extraction and/or plated on gelatin-coated dishes in serum/LIF conditions.

For FC-IS, cells were re-suspended in PBS after the immunocytochemistry procedure (see Immunocytochemistry section) and samples with no primary antibody were used as negative controls.

RNA Extraction and RT-PCR

Total RNA was extracted from 10^6 cells using High Pure RNA Isolation kit (Roche Diagnostics), with the inclusion of DNAseI treatment according to manufacturer's instructions. The first strand cDNA was synthesized from 0.5 ug of total RNA using SuperscriptII Revese Transcriptase (Invitrogen) and random hexamers. After synthesis, each cDNA was diluted 5-fold and 5 ul of diluted cDNA used in PCR reaction with gene-specific primers (Table S2). The absence of contaminating genomic DNA was confirmed for each RNA extraction by PCR amplification of *Gapdh* specific product from RT negative samples. The relative amount of each transcript was normalized to the level of *Gapdh*.

Quantitative PCR reactions were carried out in ABI Prism 7000 detection system (Applied Biosystems) in a 20 ul volume containing Itaq SYBR green Supermic mix (Bio-Rad) and 0.2 mM of each primer. Forty cycles were performed, each consisting of 10s denaturation at 95°C and 1 min primer annealing and elongation at the respective primers annealing temperature. The specificity of the reactions was confirmed using melting curve and gel electrophoresis analysis to confirm the presence of a single band. Control assays containing no templates were also performed. $2^{-\Delta\Delta Ct}$ method was used to calculate expression levels [37] and results were subsequently analysed for statistical significance using a t-test.

Northern Blot Analysis

Total RNA (5μg/lane) was separated on formaldehyde gel electrophoresis and transferred onto Hybond N+ membrane (GE Healthcare) in 10×SSC, UV-crosslinked and hybridized at 42°C with [α-32P]- dCTP (3000 Ci/mmol; Perkin-Elmer/NEN)-labeled DNA probe in Ultrahyb® ultrasensitive hybridization buffer (Ambion). Radioactive probes were cleaned from unincorporated radionuclide on Ilustra Microspin G-50 columns (GE Healthcare) prior to hybridization. In all experiments, probes with greater than 0.5×10^9 dpm/μg activity were used. Approximate lengths of different mRNAs were estimated by comparison with the migration distance of rRNAs. (The same blots were hybridized sequentially with probes for different transcripts, after stripping in 0.5% SDS at 65°C).

Protein and RNA Half-lives

To determine protein half-life, protein synthesis was inhibited by treating cells with 100 ug/ml of cycloheximide [38] and aliquots were taken at different time points (0 h, 0.5 h, 1 h, 3 h and 6 h). 6 h DMSO treated cells were used as a control and the decay of the targeted proteins was determined by western blot analysis.

To determine RNA half-life, transcription was blocked by treating ES cells with 5 ug/ml of actinomycin D [39] and samples were collected at different time points (0 h, 0.25 h, 0.5 h, 0.75 h, 1 h, 2 h, 3 h, 4 h, 5 h and 6 h). 6 h DMSO treated cells were used as a control. Total RNA was extracted, cDNA was synthesised and quantitative PCR was performed as described in the "RNA extraction and RT-PCR" Materials and Methods section.

Karyotypic Analysis

Karyotypic analyses were performed for E14tg2a, Nd and TNG-A ES cell lines. Mitotic chromosome preparations were carried out using standard procedure available at the Jackson laboratory website [40]. Metaphase chromosomes were stained with DAPI, photographed and counted using ImageJ software. At least 60 metaphase nuclei were examined for each cell line (Figure S3).

Supporting Information

Figure S1 ES cell lines characterization: E14tg2a, TNG-A and transgenic clones. (A) Bright field images of E14tg2a, TNG-A and BAC-transgenic clones grown in serum/LIF conditions. Cell growth, measured as fold increase, is shown in brackets. (B) Representative FC histograms of VNP expression for E14tg2a (negative control, grey peak), TNG-A and transgenic clones (black lines) grown in serum/LIF. Reporters expression averages and standard deviations of at least 2 independent experiments are depicted on each graph for the corresponding cell line.
(TIF)

Figure S2 Copy number of integrated BAC in Nd ES cells. (A) Relative quantities of BAC (pBAC) and of Nanog (pNanog) were measured on a dilution series of DNA plasmids containing each sequence. Threshold cycle (Ct) values were plotted against log-transformed concentrations of plasmid DNA (n = 2). Trend lines were inserted and used to obtain values for slope (y = *slope*.x+*y-intercept*) and correlation coefficients (R^2). (B) Quantities of pBAC relative to pNanog were determined for the wild-type E14tg2a ES cell line and for the Nd ES cell clone (n = 2). Plot shows that, as expected, no pBAC integration exists in wild-type ES cells, while the Nd clone has one copy of pBAC per two of pNanog.
(TIF)

Figure S3 Karyotypic analysis of Nd, E14tg2a and TNG-A ES cell lines. (A) Percentages of diploid (n = 40) and aneuploid metaphases for Nd cells at passage 8, E14tg2a cells at passage 22 and TNG-A cells at passage 40. (B) Total number of metaphases counted. (C) Microphotographs of typical diploid metaphases for each cell line. Scale bar = 10 μm.
(TIF)

Figure S4 Representative dot blots for Nanog:VNP expression after re-plating of Nd ES cells subpopulations in serum/LIF. FACS sorted populations of Nd ES cells (VNP_L and VNP_H) were replated in serum/LIF and reporter's expression was measured for four consecutive days (see Figure 5B). After 2–4 days, heterogeneity is re-established and expression of Nanog:VNP is similar between the three populations, either derived from the sorted VNP_L and VNP_H subsets, or from the whole population ("All"). The observed rate of reversion is faster and more robust for the VNP_H than for the VNP_L subpopulations (respectively, 2 and 4 days). E14tg2a cells were used as a negative control, to obtain the positive gate region.
(TIF)

Table S1 List of antibodies used for the immunostaining analyses.
(DOCX)

Table S2 List of gene-specific primers used in RT-PCR and primers used for BAC recombineering.
(DOC)

Table S3 Protein expression: statistical analysis. All p-values were calculated using a two-tailed distribution, two-sample equal variance t-test. Statistical significant p-values (p-value <0.05) are highlighted in bold, while non-significant differences (p-value >0.05) are depicted in italic. (A) p-values for each cell line grown in serum/LIF vs 2i media. In both culture conditions, similar OCT4 and SOX2 expressions are observed for all cell lines, while statistically significant differences exist in NANOG expression levels. For Nd ES cells, statistically significant differences are additionally observed in VNP expression. (B) p-values for Nd vs E14tg2a cells. In all analysis, no statistically significant difference is observed between Nd and E14tg2a ES cells. (C) p-values for Nd vs TNG-A cells. Statistically significant differences are only observed in VNP/GFP expression when Nd and TNG-A cells are grown in serum/LIF conditions.
(DOCX)

Acknowledgments

We would like to thank Austin Smith and Meng Li (Cambridge, UK) for ES cell lines, Ana Luisa Caetano (IMM, Lisbon, Portugal) for FACS sorting and Ana Margarida Cristovão for BAC recombineering. We also thank Catarina Ramos and Ana Guedes for critical reading of the manuscript.

Author Contributions

Conceived and designed the experiments: EA EB DH. Performed the experiments: EA EB. Analyzed the data: EA EB DH. Wrote the paper: EA DH.

References

1. Potten CS, Loeffler M (1990) Stem cells: attributes, cycles, spirals, pitfalls and uncertainties. Lessons for and from the crypt. Development 110: 1001–1020.
2. Morrison SJ, Shah NM, Anderson DJ (1997) Regulatory mechanisms in stem cell biology. Cell 88: 287–298.
3. Martin GR (1981) Isolation of a pluripotent cell line from early mouse embryos cultured in medium conditioned by teratocarcinoma stem cells. Proceedings of the National Academy of Sciences of the United States of America 78: 7634–7638.
4. Evans MJ, Kaufman MH (1981) Establishment in culture of pluripotential cells from mouse embryos. Nature 292: 154–156.
5. Smith AG (2001) Embryo-derived stem cells: of mice and men. Annu Rev Cell Dev Biol 17: 435–462.
6. Ying Q-L, Wray J, Nichols J, Batlle-Morera L, Doble B, et al. (2008) The ground state of embryonic stem cell self-renewal. Nature 453: 519–523.
7. Silva J, Smith A (2008) Capturing pluripotency. Cell 132: 532–536.
8. Niwa H (2007) How is pluripotency determined and maintained? Development 134: 635–646.
9. Young RA (2011) Control of the embryonic stem cell state. Cell 144: 940–954.
10. Jaenisch R, Young R (2008) Stem cells, the molecular circuitry of pluripotency and nuclear reprogramming. Cell 132: 567–582.
11. Thomson M, Liu SJ, Zou L-N, Smith Z, Meissner A, et al. (2011) Pluripotency factors in embryonic stem cells regulate differentiation into germ layers. Cell 145: 875–889.
12. Loh KM, Lim B (2011) A precarious balance: pluripotency factors as lineage specifiers. Cell Stem Cell 8: 363–369.
13. Ishigaki Y, Li X, Serin G, Maquat LE (2001) Evidence for a pioneer round of mRNA translation: mRNAs subject to nonsense-mediated decay in mammalian cells are bound by CBP80 and CBP20. Cell 106: 607–617.
14. Toyooka Y, Shimosato D, Murakami K, Takahashi K, Niwa H (2008) Identification and characterization of subpopulations in undifferentiated ES cell culture. Development 135: 909–918.
15. Singh AM, Hamazaki T, Hankowski KE, Terada N (2007) A heterogeneous expression pattern for Nanog in embryonic stem cells. Stem Cells 25: 2534–2542.
16. Chambers I, Silva J, Colby D, Nichols J, Nijmeijer B, et al. (2007) Nanog safeguards pluripotency and mediates germline development. Nature 450: 1230–1234.
17. Osorno R, Chambers I (2011) Transcription factor heterogeneity and epiblast pluripotency. Philosophical Transactions of the Royal Society B: Biological Sciences 366: 2230–2237.

18. Mitsui K, Tokuzawa Y, Itoh H, Segawa K, Murakami M, et al. (2003) The homeoprotein Nanog is required for maintenance of pluripotency in mouse epiblast and ES cells. Cell 113: 631–642.
19. Chambers I, Colby D, Robertson M, Nichols J, Lee S, et al. (2003) Functional expression cloning of Nanog, a pluripotency sustaining factor in embryonic stem cells. Cell 113: 643–655.
20. Copeland NG, Jenkins NA, Court DL (2001) Recombineering: a powerful new tool for mouse functional genomics. Nat Rev Genet 2: 769–779.
21. Nagoshi E, Saini C, Bauer C, Laroche T, Naef F, et al. (2004) Circadian gene expression in individual fibroblasts: cell-autonomous and self-sustained oscillators pass time to daughter cells. Cell 119: 693–705.
22. Vilas-Boas F, Fior R, Swedlow JR, Storey KG, Henrique D (2011) A novel reporter of notch signalling indicates regulated and random notch activation during vertebrate neurogenesis. BMC Biol 9: 58.
23. Abranches E, Silva M, Pradier L, Schulz H, Hummel O, et al. (2009) Neural differentiation of embryonic stem cells in vitro: a road map to neurogenesis in the embryo. PLoS ONE 4: e6286.
24. Kalmar T, Lim C, Hayward P, Muñoz-Descalzo S, Nichols J, et al. (2009) Regulated fluctuations in nanog expression mediate cell fate decisions in embryonic stem cells. PLoS Biol 7: e1000149.
25. Kunath T, Saba-El-Leil MK, Almousailleakh M, Wray J, Meloche S, et al. (2007) FGF stimulation of the Erk1/2 signalling cascade triggers transition of pluripotent embryonic stem cells from self-renewal to lineage commitment. Development 134: 2895–2902.
26. Stavridis MP, Lunn JS, Collins BJ, Storey KG (2007) A discrete period of FGF-induced Erk1/2 signalling is required for vertebrate neural specification. Development 134: 2889–2894.
27. Eblaghie MC, Lunn JS, Dickinson RJ, Munsterberg AE, Sanz-Ezquerro JJ, et al. (2003) Negative feedback regulation of FGF signaling levels by Pyst1/MKP3 in chick embryos. Curr Biol 13: 1009–1018.
28. Kawakami Y, Rodriguez-Leon J, Koth CM, Buscher D, Itoh T, et al. (2003) MKP3 mediates the cellular response to FGF8 signalling in the vertebrate limb. Nat Cell Biol 5: 513–519.
29. Rathjen J, Lake JA, Bettess MD, Washington JM, Chapman G, et al. (1999) Formation of a primitive ectoderm like cell population, EPL cells, from ES cells in response to biologically derived factors. J Cell Sci 112 (Pt 5): 601–612.
30. Soncin F, Ward CM (2011) The Function of E-Cadherin in Stem Cell Pluripotency and Self-Renewal. Genes 2: 229–259.
31. Wang Y, Mah N, Prigione A, Wolfrum K, Andrade-Navarro MA, et al. (2010) A Transcriptional Roadmap to the Induction of Pluripotency in Somatic Cells. Stem cell reviews.
32. Teo AKK, Arnold SJ, Trotter MWB, Brown S, Ang LT, et al. (2011) Pluripotency factors regulate definitive endoderm specification through eomesodermin. Genes & development.
33. Plusa B, Piliszek A, Frankenberg S, Artus J, Hadjantonakis AK (2008) Distinct sequential cell behaviours direct primitive endoderm formation in the mouse blastocyst. Development 135: 3081–3091.
34. Chazaud C, Yamanaka Y, Pawson T, Rossant J (2006) Early lineage segregation between epiblast and primitive endoderm in mouse blastocysts through the Grb2-MAPK pathway. Developmental Cell 10: 615–624.
35. Wray J, Kalkan T, Smith AG (2010) The ground state of pluripotency. Biochem Soc Trans 38: 1027–1032.
36. Chan W, Costantino N, Li R, Lee SC, Su Q, et al. (2007) A recombineering based approach for high-throughput conditional knockout targeting vector construction. Nucleic Acids Res 35: e64.
37. Pfaffl MW (2001) A new mathematical model for relative quantification in real-time RT-PCR. Nucleic Acids Res 29: e45.
38. Zhou P (2004) Determining protein half-lives. Methods Mol Biol 284: 67–77.
39. Sobell HM (1985) Actinomycin and DNA transcription. Proc Natl Acad Sci U S A 82: 5328–5331.
40. Akeson EC, Davisson MT (2001) Mitotic chromosome preparations from mouse cells for karyotyping. Curr Protoc Hum Genet Chapter 4: Unit4 10.
